# The Influence of Demographic Characteristics on Student Academic Performance in University Admission Tests

**DOI:** 10.11621/pir.2025.0405

**Published:** 2025-12-15

**Authors:** Bolatbek Abdrasilov, Morteza Charkhabi, Lyazzat Shinetova, Naghi Radi Afsouran, Elena Kardanova

**Affiliations:** a National Testing Center, Astana, Kazakhstan; b HSE University, Moscow, Russia; c University of Guilan, Rasht, Iran

**Keywords:** demographic characteristics, academic performance, gender differences, language differences, unified national test

## Abstract

**Background.:**

Psychological attention to the role of academic performance in university admission tests is still growing. This attention includes both the psychometric characteristics of these tests and the cognitive and non-cognitive factors that may affect performance in admission tests.

**Objective.:**

This study aims to assess the stability and changes in the academic performance of students across two consecutive admission tests. It also aims to examine how participants’ genders and languages can relate to student academic performance.

**Design.:**

To test the research hypotheses, we sampled a large group of school graduates (n = 25.563) who applied and took two national university admission tests in 2024 in Kazakhstan.

**Results.:**

The results of the Pearson correlation revealed that there is a positive association between the academic performance of applicants in two consecutive national admission test scores (r = .913). The paired t-test results suggested that they were statistically signiicant, with the largest increase in overall scores (*AM* = .96). Gender was found to affect the academic performance such that females outperformed males (85.6% vs. 64.6% pass rates), showing stronger effect sizes. There were also statistical differences between the two groups of Russian and Kazakh test takers; however, this difference was not practically significant.

**Conclusion.:**

These findings suggest that demographic characteristics of applicants can considerably influence the university admission results, and it is recommended that test developers be attentive to this issue. Academic performance showed strong stability over time, especially in STEM subjects, with significant but modest overall improvements across subjects.

## Introduction

University admission tests are not merely tools for examining the minimum required knowledge of applicants before enrollment in higher education institutions or for choosing candidates who can successfully complete a university program without academic failure (Tinto, 1975), but are also considered tools that contribute to a nation’s future success and economic growth by selecting the most qualified candidates for future job vacancies (McGrath et al., 2015; Charkhabi et al., 2025). For this reason, the world’s top universities pay particular attention to their admission tests and regularly assess the efficacy and reliability of these tests over time (Zwick, 2017; Davies et al., 2022). For example, 31 out of 38 member states of the Organization for Economic Co-operation and Development conduct national exams for university admission (OECD, 2020). Similarly, 14 out of the 15 former Soviet republics use university admission tests (Bethell & Zabulionis, 2012).

Although test developers attempt to use advanced methodological and psychometric techniques to develop valid and reliable admission tests that can differentiate between low- and high-performing candidates, these attempts may fail if they do not consider factors that may affect the fairness, biases, and effectiveness of these tests when they are applied to large populations. These findings should press psychome-tricians to carefully reflect and identify any sources of assessment bias. Studies have shown that socioeconomic factors (McManus et al., 2013; Zwick, 2017), cultural and lingual factors (Santelices & Wilson, 2010), and psychological factors such as test anxiety (Von der Embse, 2018) are factors that may affect applicants’ performance in admission tests. Recent studies have also pointed to the role of gender in achieving success in admission tests (Charkhabi et al., 2025; Niessen et al., 2019).

In Kazakhstan, university admission is based on a selective admission system (Abdrasilov et al., 2025), and the admission test is called the Uniied National Test (UNT), which was introduced in 2004. This test aims to enhance the overall quality of education, objectively assess high school graduates’ knowledge, identify the most talented candidates for university education, and ensure the fair distribution of state scholarships.

Every student is allowed to make multiple attempts on this test, but only on different occasions. Typically, a university applicant can take this test up to ive times a year at the centers introduced by the Ministry of Education. UNT scores are the only criterion for selecting and admitting candidates to Kazakh higher education institutions. According to statistics, between 2004 and 2024, an average of 126,300 applicants took the UNT annually (Abdrasilov et al., 2024). Overall, in the 20 years of history of UNT assessments, more than 2 million applicants have taken the exam — equivalent to approximately 10% of the country’s current population. For many candidates a successful high score on UNT means access to university education, future employment, and overall economic prosperity. The test consists of 120 closed-ended questions, with a maximum score of 140 points distributed across ive subjects. It lasts 240 minutes, and the results are available immediately, as scoring is done automatically. Since the test can be taken in either Kazakh or Russian by male and female candidates, there is a lack of information regarding how sociodemographic characteristics might affect test performance. As such, this study aims to examine the results of admission tests across applicants’ gender and test language among participants who took two consecutive UNTs.

### A Review of Current Literature

Admission tests vary from one country to another in terms of degree of standardization; whether they are mandatory or optional; whether they focus on content or skills; their frequency and retake policies; and if they are single admission criteria or part of a more comprehensive admission system. Some countries, in addition to standardized admission tests, offer exams specifically designed for admission to certain disciplines. For example, there are well-known standardized admission exams in Germany, and the UK — namely, the *Abitur* and *A-levels* — but medical school applicants are required to take discipline-specific exams, such as the *Test für Medizinische Studiengänge* (TMS) in Germany, and the United Kingdom requires the *Clinical Aptitude Test* (UKCAT) (Meyer et al., 2019). Moreover, some countries have unique admission practices rather than standardized procedures. For example, in Denmark, students are accepted into tertiary education based on a holistic approach. Furthermore, in addition to admission exams, in Denmark students are required to provide a resumé, examples of written assignments, and participate in interviews — all elements traditionally associated with graduate programs (Gandil & Leuven, 2022). In Switzerland, the certiicate of school completion and admission to universities is based in part on the *Matura* paper — an academic paper that all students are expected to write at the end of secondary education (Hirt et al., 2021).

With this growing attention to admission tests, attention to the psychometric quality of these tests is also growing. This includes not only attention to the validity and reliability of tests but also any potential factors that can increase the risk of creating biases in test results. Although the primary research attempted to show the link between successful performance in these admission tests and university first-year GPA (Bai et al., 2014; Migliaretti et al., 2017), some argue that high school grades are a more strongly correlated factor in determining college performance than test scores (Zwick, 2019). New studies suggested a combination of high school grades and university admission tests as a balanced approach that most accurately predicts university success (Burton & Ramist, 2001; Kobrin & Patterson, 2008; Ferräo & Almeida, 2018). Additionally, researchers have tried to identify factors that influence a candidate’s performance in these admission tests. For example, some researchers have argued that admission tests may overlook the role of those socioeconomic and demographic characteristics of candidates that can substantially affect test results. They have referred to factors such as parents’ level of education or wealth, race and ethnicity, gender, and place of residence, among others (*e.g.,* Rothstein, 2004; Atkinson & Geiser, 2009). These factors may influence the fairness of admission tests for candidates with diferent socio-demographic characteristics.

Despite the UNT’s more than two-decade-long history, these factors remain largely unaddressed in the pedagogical assessment literature. Most national scholars discuss the UNT from a primarily descriptive perspective, ofering little analytical evaluation or scholarly insight. Among the limited existing literature on the subject, one of the possible drawbacks of the UNT that has attracted scholarly attention is the role of private tutoring as a signiicant socioeconomic factor inluencing results (Smagulova et al., 2025; Hajar & Abenova, 2021). One of the most common approaches to studying the UNT is through the lens of evaluating demographic factors — such as gender, place of origin, and type of residence, or language of examination — all variables that have been found to affect test outcomes (Mingisheva, 2023; Amangeldiyeva, 2024; Shabdenova and Satybayeva, 2024). These studies tend to highlight patterns in high-stakes exams — such as higher performance among urban students compared to their rural peers, or better results by females than males in most subjects — without providing theoretical frameworks to explain these trends.

One demographic factor that may influence outcomes is gender. Although studies in the 20th century suggested that traditionally, men perform better in STEM in comparison to women, in recent years this gap has been disappearing (Charlesworth & Banaji, 2019). At the same time, the differences remain significant if we look at high-performers in the upper edge of the scores, as we would observe overrepresen-tation of males in subjects such as math and physics, while females may have some advantage in language and creative disciplines (Zawistowska, 2017; Charlesworth & Banaji, 2019).

The difference in performance between men and women is attributed to different reasons. For example, test-taking behavior may explain the gender gap, as some studies show that female applicants more often use the strategy of random guessing and demonstrate higher levels of anxiety during the tests, while high-scoring male applicants are less anxious and possess greater motivation (Stenlund et al., 2017). Similarly, it is argued that women in STEM often have lower self-confidence, which can affect their performance (Gándara & Silva, 2016). Also, a recent study suggested a gender gap in STEM in Kazakhstan may be caused by schoolteachers, as they may hold biased gender-related perspectives, assuming female students to be inferior in subjects such as physics and computer science (Durrani & Kataeva, 2025). However, effects on national admission tests that correspond with these observations have not been previously studied in Kazakhstan. Thus, the first aim of this study is to investigate the influence of gender on two consecutive UNT results, as formulated in the hypothesis below:

*Hypothesis 1.* There is a significant difference between male and female candidates in UNT results

Another potential socio-demographic factor that may influence applicants’ performance in admission tests is the language of the test or the language of the test-taker. Th ere is evidence that shows that test language can influence admission results (Santelices, & Wilson, 2010). Th ere are countries where citizens speak multiple languages, and the test developers consider language differences in preparing their educational assessments. For example, in countries like Canada or Switzerland, which have a plurality of languages, provinces or cantons with their own languages also have their own educational policies, making direct language-based comparisons impossible. Another potential comparative example could be the Republic of South Africa, which has twelve official languages, which causes an educational conundrum (Mphasha et al., 2022; Munyai, 2024). However, most subjects in its *National Senior Certificate* admission test are offered only in English or Afrikaans. As a result, test outcomes are inluenced not by language choice but by language proiciency, since applicants from minority language groups must take the test in a language that is not their mother tongue.

Language factors pertaining to the UNT are unique. It is a standardized test, and regardless of the language chosen (Kazakh or Russian) by applicants, the results are not expected to vary — an issue that will be examined here. What is particularly notable is that, despite Kazakhstan having two official languages, it remains a unitary state with a single educational policy. Therefore, the test is identical in content regardless of the language in which it is taken. Therefore, comparing the two Kazakh-stani UNT tests is implausible, as both Kazakh- and Russian-speaking applicants can take the test in their preferred languages. However, currently, there is no study to show that the results of this test in both languages are similar. Thus, this study aims to compare the UNT results across Kazakh and Russian test takers, as reflected in the following hypothesis:

*Hypothesis 2.* There is a significant difference between Kazakh and Russian test-takers in UNTs results

## Methods

### Participants

In the present study, our focus is solely on applicants who took the UNT twice between May 16 and July 5, 2024, as part of the application process for tuition-free admission. This is the main examination period, attracting the highest number of participants annually, with more than 181.000 applicants in 2024. Specifically, we focus on applicants who selected the math and physics combination. This was the most popular choice among high school graduates in 2024, with 25.563 applicants (males: 18.495; females: 7068) who took the exam twice choosing this combination. In Kazakhstan, students usually finish high school at the age of 17, and they are then eligible to take the university admission test. As a result, the study is expected to yield more valid conclusions. Most importantly, however, this choice is rationalized by special attention paid to STEM subjects in Kazakhstan; due to the country’s aspiration to develop alternative paths for economic growth, reduce its dependence on natural resources, and remain competitive in the rapidly developing global technological and industrial market. In the context of the Fourth Industrial Revolution, amid a rapidly globalizing and digitalizing world, Kazakhstan aims to adapt its educational policy to keep pace with economic and social developments and to produce the necessary cadre of experts and leaders (Karayev & Duisenova, 2024).

### Procedure

The national test is conducted from May to July. During this period, applicants can take the test twice, with only the better result counted as final. In addition, high school students can take the exam in January, March, and August; however, these sessions fall outside the competition for state scholarships, and those taking the exam during these periods are eligible only for tuition-based education. These sessions are also open to individuals who previously failed the exam and wish to try again. Currently, the UNT can be taken at 46 regional testing centers across the country with each equipped with computers and two surveillance cameras per candidate to ensure transparency and prevent violations. Each participant undergoes biometric identii-cation by presenting their ID to a camera, which veriies that the individual taking the exam matches the registered candidate. The test includes close-ended item types such as multiple-choice questions (with single or multiple correct answers), context-based questions, and matching tasks. The duration of the test is 240 minutes, during which applicants are free to navigate between sections within the online platform according to their personal preferences, as no time restrictions are applied to individual subject areas. Scoring is conducted automatically, and results are available immediately upon completion of the exam. Recipients of government scholarships are selected based on UNT results for enrollment in universities across Kazakhstan. This includes local branches of international universities. These branch campuses, in addition to their own admission criteria, require speciic UNT scores for scholarship-based admission.

Concerning the choice of electives, for example, those interested in studying the fundamentals of law and economics, philosophy, or religious studies need to choose a combination of history and geography as their elective subjects. Similarly, the combination of math and physics are necessary for applicants who wish to specialize in nuclear physics, automation and control, astronomy, geological and geophysical exploration for mineral deposits, mining, or other ields related to these elective subjects. For each discipline that candidates intend to pursue in higher education, a spe-ciic combination of test subjects are required.

### Measures

The UNT consists of 120 questions, with a maximum score of 140 points. The test covers three core subjects — reading literacy (10 questions), mathematical literacy (10 questions), and the history of Kazakhstan (20 questions), and two elective subjects based on the applicant’s intended specialization (40 questions each). All items (test questions) that comprise the core subjects along with the first 30 items of elective subjects are scored dichotomously, while the last 10 items of elective subjects are scored polytomously with a maximum of 2 points. A minimum set of scores are assigned for each subject: at least 5 points in the history of Kazakhstan and in the specialized subjects, and at least 3 points in reading literacy and mathematical literacy. Passing score ranges from 50 to 75, depending on the university, subject, and form of study. The minimum passing threshold of 50 is set by the Ministry of Science and Higher Education by law, but the criteria or justification for this threshold has not been conveyed. The test can be taken in three languages including, Kazakh, Russian, and English. From 2021, up to five test attempts can be taken per year (Abdrasilov et al., 2025). In 2021, the Unified National Test transitioned to a fully computer-based format.

### Data analysis

The data analysis followed several steps, including data cleaning, primary data analysis, and inal data analysis. First, the data was checked for any possible outliers and missing cases. Participants with invalid or unspeciied demographic status were discarded. Second, we used SPSS, JASP, and JAMOVI programs to draw correlation matrices between two consecutive UNT results. We also used the same statistical programs to compare the results across diferent genders and test languages using paired t-tests and chi-square analysis.

## Results

### Descriptive Statistic

*Table 1* presents descriptive statistics for subjects’ assessment scores in two consecutive UNTs across two time points (T1 and T2). As the table shows, at T1, the mean overall score was 64.62 (*SD* = 22.31), but it increased slightly to 65.58 (*SD* = 22.97) at T2. Among the subjects, history scores improved from time 1 (9.90) to time 2 (10.21). In addition, math scores had the highest scores at both time 1 (22.69) and time 2 (23.07), with the highest variability (*SDs* ~ 10.86-11.08) among all subjects. Scores in reading literacy and math literacy remain almost identical (*e.g*., reading: T1 = 7.70, T2 = 7.79). Overall, scores for the second attempt increased over the first attempt results.

**Table 1:** Descriptive statistics of research variables in the present study (n = 25563)

Time	Variable	Range	Min	Max	Mean (SD)	Variance	Skewness (SE)	Kurtosis (SE)
T1	History	20	0	20	9.90 (3.27)	10.69	.212 (.015)	-.234 (.031)
	Reading literacy	10	0	10	7.70 (1.78)	3.18	-.895 (.015)	.735 (.031)
	Math literacy	10	0	10	6.18 (2.35)	5.54	-.248 (.015)	-.750 (.031)
	Physics	50	0	50	18.15 (8.27)	68.46	1.127 (.015)	.862 (.031)
	Math	50	0	50	22.69 (10.86)	118.04	.604 (.015)	-.701 (.031)
	Overall score	118	20	138	64.62 (22.31)	497.58	.855 (.015)	-1.270 (.031)
T2	History	20	0	20	10.21 (3.45)	11.90	.231 (.015)	-.306 (.031)
	Reading literacy	10	0	10	7.79 (1.78)	3.18	-1.010 (.015)	1.054 (.031)
	Math literacy	10	0	10	6.21 (2.39)	5.69	-.248 (.015)	-.771 (.031)
	Physics	50	0	50	18.30 (8.50)	72.32	1.159 (.015)	.881 (.031)
	Math	50	0	50	23.07 (11.08)	122.84	.572 (.015)	-.763 (.031)
	Overall score	122	16	138	65.58 (22.97)	527.71	.729 (.015)	-.247 (.031)

### Correlation results

*Table 2* presents the results of the correlation analysis of the test performance of students in different subjects across two consecutive tests. As the table shows, although there are correlations between the subjects’ scores, the strongest correlation between the paired scores is represented by the math score (*r* = .881, *p* < .001) and the physics scores (*r* = .803, *p* < .001). Additionally, there was a statistically positive association between the overall scores in Time 1 and Time 2 (*r* = .913, *p* < .001), which supports the high test-retest reliability of the overall performance as well. Consistently, in Time 1, the math scores were more strongly associated with the overall score in Time 2 (*r* = .876, *p* < .001), and physics scores also were strongly associated with the overall score in Time 2 (*r* = .811, *p* < .001). The weakest correlation size was found between scores in reading literacy in Time 1 and Time 2 (*r* = .433, *p* < .001). Additionally, we assessed the test-retest reliability using a two-way mixed-effects in-terclass correlation (ICC) for absolute agreement. The results suggested excellent test-retest reliability for the overall scores as follows: ICC (3, 1) = .91, 95% CI [.91, .92], *p* < .001.

**Table 2:** Pearson’s correlations between subject scores in the first and second UNT intake (n = 25563)

(T1)\(T2)	History	Reading literacy	Math literacy	Physics	Math	Overall
History	.529***	.339***	.418***	.486***	.496***	.569***
Reading literacy	.339***	.433***	.376***	.294***	.347***	.400***
Math literacy	.413***	.357***	.618***	.552***	.673***	.667***
Physics	.496***	.300***	.535***	.803***	_.744***_	.811***
Math	.506***	.347***	.654***	.751***	.881***	.876***
Overall	.579***	.402***	.689***	.815***	.875***	.913***

Note. ***p <.001

This pattern shows that some STEM subjects, such as math and physics, exhibit stronger association over time than non-STEM subjects such as reading literacy.

### T-test Results

The paired samples t-tests revealed statistically significant improvements across all academic domains (all ps < .05); however, effect sizes varied. The most substantial increases were observed in overall scores (*ΔM* = .96, |*d*| = .102, *t*(*df*) = -16.23, *p* < .001) and history (*ΔM* = .31, |*d*| = .096, *t*(*df*) = -15.32, *p* < .001). Math scores showed moderate improvement (*ΔM* = .37, |*d*| = .070, *t*(*df* = -11.19, *p* < .001), while reading literacy scores demonstrated a smaller, but still signiicant increase (*ΔM* = .09, |*d*| = .050, *t(df)* = -7.99, p < .001). Notably, physics (*ΔM* = .15, |*d*| = .03) and math literacy (*ΔM* = .03, |*d*| = .02) showed minimal changes, but meaningful enough to cross the threshold of significance. The pattern of results suggests domain-specific learning efects, demonstrating diferential improvement paths.

**Table 3:** Paired samples t-test results for academic performance measures (n = 25563)

Variable	Measure 1 *M (SD)*	Measure 2 *M (SD)*	*t*	*df*	*p*	Cohen’s |*d*|
History	9.90 (3.27)	10.21 (3.45)	-15.32***	25.563	< .001	.10
Reading literacy	7.70 (1.78)	7.79 (1.78)	_-_7 99***	25.563	< .001	.05
Math literacy	6.18 (2.35)	6.21 (2.38)	-2.34*	25.563	.019	.02
Physics	18.15 (8.27)	18.30 (8.50)	-4.45***	25.563	< .001	.03
Math	22.70 (10.87)	23.07 (11.08)	-11.19***	25.563	< .001	.07
Overall score	64.63 (22.31)	65.59 (22.97)	-16.23*	25.563	< .001	.10

*Note. All tests were two-tailed paired-samples t-tests. M = mean; SD = standard deviation; Cohen’s d = effect sizes.*

Following *Table 4,* the analysis revealed significant gender differences across academic measures. The paired samples t-tests revealed statistically significant improvements (*p* < .001) across nearly all subjects for both genders, shown by effect sizes (Cohen’s d). Among female applicants (*n* = 7.068), the largest increase was related to overall scores (*ΔM* = +1.39, |*d*| = .145) and history (*ΔM* = +.45, |*d*| = .139). Math literacy showed only marginal improvement (*ΔM* = +.05, |*d*| = .028, p = .020). Male applicants (n = 18,495) demonstrated smaller changes, with significant but negligible increases in math (*ΔM* = +.31, |*d*| = .058) and overall scores (*ΔM* = +.80, |*d*| = .085). Notably, males exhibited no signiicant improvement in math literacy (*ΔM* = +.02, |*d*| = .010, p = .179). According to Cohen (1988), an effect size of ~ .2 is considered a small effect, an effect size of ~ .5 is considered a medium effect, and an effect size of ~ .8 is considered a large efect. All efect sizes in *Table 4* are less than .2 and therefore represent no practical or meaningful signiicance, even while the t-test is signiicant. Note that large sample sizes may result in theoretically irrelevant differences (Lakens, 2013).

**Table 4:** Paired samples t-test results by gender across two consecutive UNTs (n= 25564)

Gender	Measure	First Test *M (SD)*	Second Test *M (SD)*	*t*	*Df*	*p*	Cohen’s |*d*|
Female	History	10.79 (3.28)	11.24 (3.53)	–11.681	7.067	< .001	.139
(*n*= 7.068)	Reading literacy	8.12 (1.53)	8.22 (1.52)	–4.759	7.067	< .001	.057
	Math literacy	6.73 (2.21)	6.78 (2.22)	–2.321	7.067	.020	.028
	Physics	20.73 (8.86)	20.96 (9.13)	–3.575	7.067	< .001	.043
	Math	27.01 (10.57)	27.55 (10.80)	–8.390	7.067	< .001	.100
	Overall score	73.37 (22.15)	74.76 (22.95)	–12.178	7.067	< .001	.145
Male	History	9.56 (3.20)	9.81 (3.33)	–10.803	18.494	< .001	.079
(*n*= 18.495)	Reading literacy	7.54 (1.85)	7.63 (1.85)	–6.509	18.494	< .001	.048
	Math literacy	5.98 (2.37)	6.00 (2.41)	–1.344	18.494	.179	.010
	Physics	17.16 (7.82)	17.28 (8.02)	–2.969	18.494	.003	.022
	Math	21.05 (10.52)	21.36 (10.71)	–7.937	18.494	< .001	.058
	Overall score	61.28 (21.45)	62.08 (21.99)	–11.527	18.494	< .001	.085

*Note. All tests were two-tailed paired-samples t-tests. M = mean; SD = standard deviation; Cohen’s d = effect sizes.*

As *Table 5* shows, the paired samples t-tests revealed statistically signiicant improvements (*p* < .05) across most measures for both language groups; however, the effect sizes (Cohen’s d) differed across groups. Kazakh-speaking applicants (*n* = 21.482) showed significant improvements in history (*ΔM* = +.30, |*d*| = .092), reading literacy (*ΔM* = +.10, |*d*| = .053), and overall scores (*ΔM* = +.89, |*d*| = .094), but exhibited no improvement in math literacy (*ΔM* = +.01, |*d*| = .005, *p* = .547). In contrast, Russian-speaking applicants (*n* = 4.078) demonstrated larger improvements in almost all subjects, with the largest effect sizes in overall scores (*ΔM* = + 1.38, |*d*| = .150) and history (*ΔM* = +.40, |*d*| = .121), but the lowest effect size in reading literacy (*ΔM* = +.10, |*d*| = .052). According to Cohen (1988), an effect size (d) of approximately .2 represents a *small* effect, .5 a *medium* effect, and .8 a *large* effect. As shown in *Table 5,* all observed effect sizes within the table fall below the .2 threshold (d < .2), which indicates a negligible or trivial practical signiicance despite the signiicance t-test results. This is consistent with the idea that large sample sizes may result in theoretically irrelevant differences (Lakens, 2013).

**Table 5:** Paired samples t-test results by language across two consecutive UNTs (n= 25563)

Language Group	Measure	First Test *M (SD)*	Second Test *M (SD)*	*t*	df	*p*	Cohen’s |*d*|
Kazakh	History	9.94 (3.32)	10.24 (3.51)	-13.31	21.481	< .001	.092
(n= 21.482)	Reading literacy	7.75 (1.79)	7.85 (1.78)	-7.29	21.481	< .001	.053
	Math literacy	6.12 (2.37)	6.13 (2.40)	-0.60	21.481	.547	.005
	Physics	18.19 (8.46)	18.31 (8.68)	-3.30	21.481	.001	.023
	Math	22.17 (10.72)	22.53 (10.94)	-9.93	21.481	< .001	.067
	Overall score	64.17 (22.53)	65.06 (23.19)	-13.61	21.481	< .001	.094
Russian	History	9.66 (3.00)	10.06 (3.11)	-7.77	4.077	< .001	.121
(n= 4.078)	Reading literacy	7.41 (1.73)	7.51 (1.76)	-3.39	4.077	.001	.052
	Math literacy	6.51 (2.27)	6.65 (2.26)	-4.68	4.077	< .001	.071
	Physics	17.92 (7.20)	18.22 (7.49)	-3.60	4.077	< .001	.058
	Math	25.49 (11.21)	25.92 (11.39)	-5.31	4.077	< .001	.082
	Overall score	66.98 (20.91)	68.36 (21.57)	-9.56	4.077	< .001	.150

For further analysis, a chi-square test was performed, and it similarly revealed signiicant gender diferences in test performance across both measures (T1 and T2), with female applicants consistently outperforming males. Chi-square tests confirmed the statistical significance of this gender difference for T1 (χ^2^[1] = 1097.62, *p* < .001, φ = .207) and T2 (χ^2^[1] = 1080.58, *p* < .001, φ = .206), with small effect sizes consistent across Pearson, continuity-corrected, and likelihood ratio tests (all ps < .001). Fisher’s exact tests further validated these fi ndings (*p* < .001). The similar effect sizes across both test measures suggest stable, systemic gender-based performance diferences rather than test-speciic variations.

Also, another chi-square test was performed, and the results, similar to the t-test, revealed significant differences in test performance between Kazakh- and Russian-speaking applicants across both test measures (T1 and T2). Although the pass rates between Russian-speaking applicants and their Kazakh-speaking peers differed, Chi-square tests suggested statistical significance for this language-based difference in both T1 (χ^2^[1] = 132.56, *p* < .001, φ = -.072) and T2 (χ^2^[1] = 106.61, *p* < .001, φ = -.065). Consistent with Cohen (1988) and Kim (2017), a chi-square effect size of less than .1 is considered practically non-significant, .1 is considered a small effect, .5 is considered a medium effect, and .8 is considered a large effect size for this test. As both Phi (φ) values were less than .1, we can conclude that none of these effect sizes are practically significant. The negative phi values reflect the categorical coding of language groups, while the consistency of results across likelihood ratio and Fisher’s exact tests (all ps < .001) verify the robustness of the findings.

## Discussion

The findings of this study provide new insights regarding Unified National Testing (UNT) in Kazakhstan that can be used by those countries that use a selective admission test to choose qualified students for university enrollment. These insights can be viewed and discussed from two standpoints. First, insights regarding the consistency of UNT results are important for both applicants and higher education institutions. Second, insights regarding the demographic characteristics (i.e., gender differences and test language) of applicants, may inluence the stability and consistency of the test in Kazakhstan. By interpreting these results through the lens of existing literature, we can compare the indings with previously conducted studies in other countries and better understand the underlying mechanisms that affect educational equity and policy in higher education institutions.

### UNT Consistency and Subject-Specific Performance

As noted earlier, higher education institutions regularly assess their admission tests’ quality to check and promote the stability and consistency of scoring outcomes. This is a more crucial issue regarding national admission tests, where many applicants compete to gain a better score and subsequently enroll in a more prestigious university. The findings of this study demonstrate that there are consistent and relatively stable results between two consecutive UNT results. More speciically, the stable and high correlation between math and physics scores across test measures highlighted that this stability and consistency is higher in STEM subjects (Peresetskiy & Davtyan, 2011; Bai et al., 2014). This is consistent with studies on other national exams, like the Uniied State Exam (USE) in Russia, where math scores were introduced to have higher mean scores across some faculties (Khavenson & Solovyova, 2014). The weaker correlations in history and reading literacy scores over two measures may be due to the subjective nature of these subjects in understanding and interpreting the written material submitted by students (Stemler, 2012). This may motivate UNT developers to pay more attention on assessing critical thinking by diversifying from measures of simple memorization or utilizing context-related questions (OECD, 2020).

In summary, the findings can contribute to assessment improvements that account for both psychometric and psychological factors. Psychometrically, the observed reliability of the UNT supports its construct validity as a standardized measure of the cognitive ability of applicants for university admission. However, psychologically, the practice effect caused by possible situational variables (e.g., test repetition, reduced test anxiety, improved familiarity with the test) may inluence test performance more than learning itself (Malespina, & Singh 2022; Von der Embse et al, 2018; Stenlund et al., 2017). Therefore, the distinction between test performance and academic preparedness remains a concern in validating university admission tests (Burton & Ramist, 2001; Kobrin & Patterson, 2008; Zwick, 2019).

### Gender differences in test performance

The findings also consistently revealed that female applicants outperformed in all subjects, including math and physics (85.6% pass rate vs. 64.6% for males) that are traditionally known as male-dominated subjects. The finding differs to some previous studies that showed males perform better in STEM (Charlesworth & Banaji, 2019). Th is cannot be explained using test anxiety levels as previous studies show that female test takers show higher test anxiety than males, however, the female test takes are more likely to use random guessing strategy more than their male peers to manage this anxiety and pass the admission test (Stenlund et al., 2017), This may give them an ability to sustain exam pressures more efficiently. Additionally, in Kazakhstan, public incentives such as government scholarships, which utilize UNT scores, may influence and motivate female and male applicants differently (Prakhov & Yud-kevich, 2019). Additionally, results indicate that a large number of males underper-formed, particularly in math and physics, which may suggest a systematic disengagement (Gándara & Silva, 2016).

### Language-Based diferences in test performance

The findings also demonstrate a performance gap between Kazakh and Russian test takers. Although, comparing the efect sizes between the two groups suggests that this i nding is not yet practically signiicant because all efect sizes fell below the threshold (<.02), we still can explain the trivial signiicant diference between these groups. This is consistent with previous findings that suggested in multicultural countries (e.g., Sweden), the immigration status (which may indirectly include the language background of applicants) may significantly influence outcomes (WikstrÖm, 2017). One possible explanation for this is that Russian-language schools, with greater access to private tutoring, may better prepare students for the UNT test (Hajar & Abenova, 2021).

The chi-square findings also consistently provided insights regarding the large difference between genders and language groups in UNT performance. The 21% diference in pass rates across genders (85.6% vs. 64.6% for female and male test takers respectively) demonstrates a signiicant diference in outcomes. Although other such studies have reported similar i ndings in Western admission tests (Charlesworth & Banaji, 2019), this gap seems to be higher in Kazakhstan. As the gap, with high and significant effect sizes (Phi1 = .207 & Phi2 = .206), remains stable across two tests (regardless of testing format or policy changes), one can conclude that the gender efect is not a temporary issue but rather a stable feature of the educational system in Kazakhstan. Furthermore, the consistency across various statistical tests (Pearson, continuity correction, likelihood ratio, and Fisher’s exact) verify that the results are robust. Similar chi-square indings regarding the role of language background were found. Compared to gender, language obtained lower effect sizes (Phi1 = .07 & Phi2 = .08), which are not practically significant (Kim, 2017). The consistency across all correlations verifies the robustness of the fi nding. The larger effect size of gender compared to the effect size of language introduces gender as a stronger predictor of UNT performance than language in Kazakhstan.

## Conclusion

This study reveals that Kazakhstan’s UNT exhibits stable psychometric properties, particularly in STEM subjects, but gender and language gaps remain issues that inlu-ence the results. However, this inluence still falls below the practical signiicance but may grow over time. Females outperformed males in all subjects, even STEM, which contradicts some global indings. While test scores improved slightly over time, the changes were small, meaning the UNT may reward test skills more than deep learning. To make the admission tests fairer, policymakers should focus on reducing bias in non-STEM questions and address the gaps between genders and language groups. The effect sizes for gender versus language suggest that gender is the stronger predictor of UNT performance.

## Limitations and suggestions

This study has some limitations. First, although the sample included two large groups of applicants based on gender and language background, the sample sizes of these groups were not equal, and that may have afected the pass rate results. We recommend future studies to examine the same results across groups with similar sample sizes. Second, despite identifying gender diferences and language diferences, relying on a correlational design does not allow us to draw causal relationships about whether these diferences originate from cultural factors, instructional quality, or confounding/unmeasured factors. Th ird, while the study suggested significant but modest improvements across test subjects, more reliable results can be made if future studies consider the academic performance of these two groups before they enroll at university (admission test results) and after enrollment in university (academic scores in different subjects). This would result in more reliable, stable and definite findings. The results of the test can also be used as a diagnostic guide to identify subjects in which a group has a lower performance and improve performance in that speciic subject within schools. As an example, this is particularly evident in subjects like literature, where Kazakh-speaking applicants showed no improvement across two consecutive admission tests.
